# Association between single nucleotide polymorphism (rs4252424) in *TRPV5 *calcium channel gene and lead poisoning in Chinese workers

**DOI:** 10.1002/mgg3.562

**Published:** 2019-01-21

**Authors:** Jiting Liu, Li Zhang, Lixia Feng, Ming Xu, Yue Gao, Peng Zhou, Zhengmin Yu, Baoli Zhu, Yan An, Hengdong Zhang

**Affiliations:** ^1^ Department of Toxicology, School of Public Health, Jiangsu Key Laboratory of Preventive and Translational Medicine for Geriatric Diseases Medical College of Soochow University Suzhou China; ^2^ Department of Occupational Disease Prevention Jiangsu Provincial Center for Disease Control and Prevention Nanjing China; ^3^ Public Health Research Institute of Jiangsu Province Nanjing China; ^4^ Wuzhong City Center for Disease Control and Prevention Wuzhong China; ^5^ Nanjing Medical University Nanjing China

**Keywords:** blood lead levels, calcium channel, leadexposure susceptibility, *TRPV5* polymorphism

## Abstract

**Background:**

Lead (Pb) is broadly used in various industries and causes irreversible damage to human tissues, organs, and systems. Studies have revealed that lead exerts toxic effects via interfering with calcium channel.

**Methods:**

In the present study, we investigated whether single nucleotide polymorphisms (SNPs) in *TRPV5*, a calcium channel‐related gene, were associated with lead exposure susceptibility. By using TaqMan SNP genotyping, we performed genotyping of eight *TRPV5 *tag‐SNPs in 1,130 lead‐exposed Chinese workers with similar lead exposure level.

**Results:**

Single nucleotide polymorphism rs4252424 was significantly associated with lead susceptibility, measured by blood lead level (BLL) (*β* = −0.069, *p*_linear_ = 0.029). However, there was no significant association between any other seven SNPs and BLL. The further expression Quantitative Trait Loci displayed that CC genotype of rs4252424 is significant associated with higher BLL than CT (*p* < 0.0001).

**Conclusion:**

We conclude that SNP rs4252424 has the potential to evaluate lead susceptibility in the Chinese occupational population, and further enhance lead exposure prevention and intervention.

## 1 INTRODUCTION

Lead (Pb) has been widely used in many industrial fields, including lead ore mining, pigment production, batteries manufacturing, and painting (Patil et al., 2007). Lead is mainly absorbed by inhalation and ingestion in forms of fumes and dusts in the air, following with diffusion into certain tissues and organs (Aviv, John, Bernstein, Goldsmith, & Spitzer, 1980; Goldstein, 1990; Muntner, He, Vupputuri, Coresh, & Batuman, 2003). Exposure to lead usually led to multi‐systemic body damage, such as hematopoietic, neurological and renal dysfunctions (Flora, Gupta, & Tiwari, 2012; Putnam, 1986). Blood lead level (BLL) accurately reflects recent lead exposure (Rabinowitz, Wetherill, & Kopple, 1976) and is considered as a valid and sensitive index to quantify lead absorption and exposure. A meta‐analysis has reported that high BLL increased the risk of lung cancer and stomach cancer (Steenland & Boffetta, 2000). Besides, a national epidemiologic study of the U.S. population showed that even low BLL (5–9 μg/dl) increased the risk of mortality from cardiovascular disease and cancer (Schrober, 2006). Because of the significant impacts of lead on health, it is critical to explore and identify potential biomarkers to predict and prevent lead‐related disease.

Previous studies have demonstrated that lead interferes with body functions through the following mechanisms: (a). Oxidative stress, involving excessive production of reactive oxygen species (ROS) and depletion of antioxidant against lead exposure (Flora et al., 2012; Flora, 2009); (b). Ionic mechanism (Flora et al., 2012; Simons, 1993). Pb^2+^ substitutes other bivalent cations, like Ca^2+^, Mg^2+^, Fe^2+^, binds to specific enzymes, and eventually causes body malfunction. Because of their similar physical and chemical properties, Pb^2+^‐Ca^2+^ interaction occurs in a wide range of physiological processes. Pb^2+^ and Ca^2+ ^compete in plasma membrane, especially for Ca^2+ ^channel and Ca^2+^ pump. Moreover, Pb^2+ ^perturbs a number of Ca^2+^‐dependent functional factors, for example, calmodulin and protein kinase C (Pounds, 1984; Simons, 1993). It has been shown that Pb^2+ ^effectively blocks voltage‐activated calcium channel currents in rat dorsal root ganglion neurons (Busselberg, Platt, Michael, Carpenter, & Haas, 1994). Additionally, lead decreases the concentration of intracellular calcium in brain capillary endothelial cells by weakening intercellular junctions (Kerper & Hinkle, 1997).

*TRPV5* (transient receptor potential cation channel subfamily V member 5, OMIM accession number is 606679), a member of transient receptor potential (TRP) cation channel subfamily V, mainly distributes in kidney, and is also found in placenta (Bernucci, Henriquez, Diaz, & Riquelme, 2006), osteoclasts (van der Eerden et al., 2005) and retinal pigment epithelium (Kennedy, Torabi, Kurzawa, Echtenkamp, & Mangini, 2010). It is responsible for active re‐absorption of Ca^2+^ across epithelium of the distal convoluted tubule (DCT) and connecting tubule (CNT). The process involves three major steps. First, Ca^2+ ^moves across apical membrane via TRPV5. Subsequently, Calbindin‐D_28k_ binds intracellular Ca^2+^ to carry it through the cytosol. Finally, Ca^2+^ is excreted via NCX1 (Na^+^/Ca^2+^ exchanger) and PMCA1b (Ca^2+^‐ATPase). These steps are regulated by 1,25‐(OH)_2_D_3_ and parathyroid hormone (PTH) (Mensenkamp, Hoenderop, & Bindels, 2007). As Pb^2+^ effectively blocks TRPV5 currents (Vennekens et al., 2001), lead could potentially interact with TRPV5 and influence Ca^2+^ re‐absorption process. Therefore, we hypothesize that *TRPV5 *gene polymorphism is potentially associated with susceptibility to lead exposure, and have conducted the study to explore this hypothesis in a Chinese occupational population with lead exposure.

## 2 MATERIALS AND METHODS

### 2.1 Study population

The study population consisted of 1,130 volunteers. They were workers from five lead‐acid battery factories in Jiangsu Province, China, and were under similar external lead exposure dose (0.017 ± 0.004 mg/m^3^) (Zhang et al., 2017). Before this study, each volunteer signed an informed consent. After initial screening, some volunteering workers were excluded from this study according to the following criteria: (a) with initially aberrant BLLs; (b) with a history of hematological disorders, liver or kidney dysfunction; (c) exposed to medicine containing lead in daily life. Then the included participants were interviewed, and their detailed demographic characteristics, occupational history, medical history, individual habits, and self‐conscious symptoms were recorded. We ranked participants’ lead exposure level based on their BLLs. This study was approved by the Ethics Committee of the Jiangsu Provincial Center for Disease Control and Prevention (approval No. 2012025).

### 2.2 BLLs determination

5‐ml venous blood collected from each participant was placed in a metal‐free vacuum blood collecting tube and stored at −4°C for 48 hr. Then, BLL was measured by atomic absorption spectrometry, using PerkinElmer model 5000 graphite furnace atomic absorption spectrophotometer (PerkinElmer, Waltham, MA, USA). More detailed information about this process is available in our previous study (Zhang et al., 2017).

### 2.3 SNP selection and genotyping

**Figure 1:**
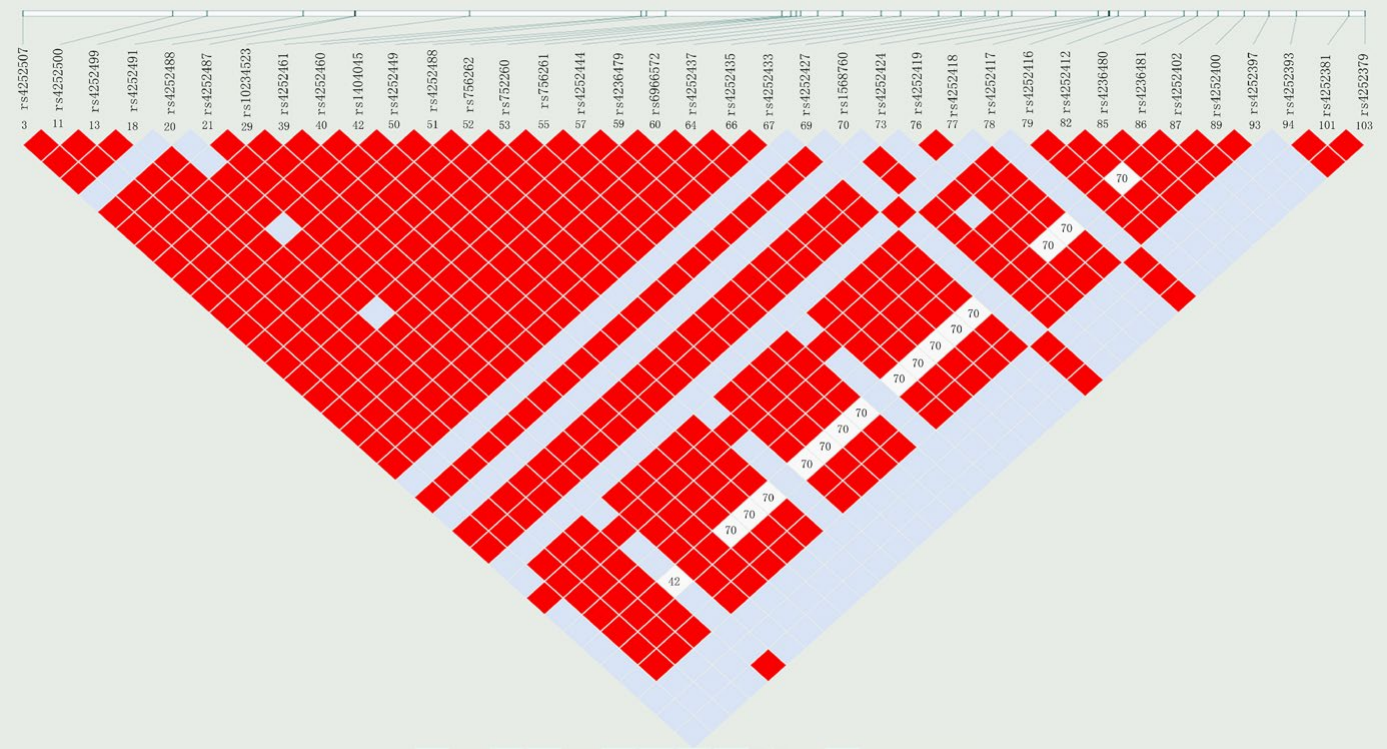
Four single nucleotide polymorphisms generated by Haploview. Shading represents the magnitude and significance of pairwise LD, with a red‐to white gradient reflecting higher to lower LD values

We conducted extensive search in HapMap and NCBI database to select SNPs in *TRPV5 *gene that had minor allele frequency of >0.05 in Han Chinese Beijing (HCB). Haploview 4.2 software was used to evaluate linkage disequilibrium between the SNPs. Finally, four SNPs were selected (*r* ^2 ^> 0.8), including rs4236480, rs4252381, rs4252424, and rs1568760 (Figure 1). Besides, four additional SNPs reported in previous studies were also included: rs4252402, rs4252416, rs4252499, and rs4252511 (Khaleel et al., 2015; Renkema et al., 2009; Southard et al., 2012). Thus, a total of eight SNPs were included in this study for genotyping and analysis.

Genomic DNA was extracted from the peripheral blood by QIAcube HT Plasticware and QIAamp 96 DNA QIAcube HT Kit (Qiagen, Dusseldorf, Germany) according to manufacturer's protocol. No external contamination had been detected. Genotypes for the eight SNPs were assayed by ABI TaqMan SNP genotyping assays (Applied Biosystems, Foster City, CA, USA). According to the manufacturer's protocols, genotyping probes, primer and extracted DNA were blended into TaqMan universal PCR master mix (Applied Biosystems). Genotyping procedures were further performed using ABI 7900 real‐time PCR system (Applied Biosystems). The corresponding genotyping data were finally analyzed via ABI 7900 System SDS software 2.4. Real‐time PCR condition was as follows: initial denaturation of 95°C for 10 min; denaturation at 95°C for 15 s; annealing at 60°C for 1 min (40 cycles of the last two steps).

### 2.4 Statistical analysis

Goodness‐of‐fit chi‐square test was used for each SNP to meet the expectation of Hardy–Weinberg equilibrium (HWE) in Chinese population. The differences of demographic characteristics in our testing group were detected by chi‐square test and student's *t* test. Continuous variables were described as the mean ± *SD*. Multiple linear regression were used to assess the association between each SNP of *TRPV5* gene and the BLL adjusted for age, gender, marriage situation, education status, smoking status and drinking status (Chu et al., 2015). *p < *0.05 was adopted as statistical significance (two‐sided). The expression quantitative trait loci (eQTL) analysis of rs4252424 was calculated by unpaired *t* test between different genotypes of polymorphism. All statistical analyses were conducted in SPSS 23.0 (Chicago, IL, USA).

## 3 RESULTS

### 3.1 1 General characteristics and clinical features of the study subjects

A total of 1,130 final participants were recruited, including 599 males and 531 females. Their age ranged between 40 and 50.98.5% were married. Education status of the participants was mainly elementary to junior middle school level (59.8%) and senior high school and above level (20.3%). 26.6% were smokers and 27.7% were drinkers. 33.5% participants never ate or drank alcohol in the workplace. The overall BLL of all participants was 386.73 ± 177.93 μg/L (Table 1).

**Table 1:** Demographic characters and BLLs of all participants

Participant characteristics	*N* = 1,130
	*N* (%)
Gender	
Male	599 (53.0)
Female	531 (47.0)
Age (years)	
[20, 30)	83 (7.4)
[30, 40)	275 (24.3)
[40, 50)	619 (54.8)
[50, 60)	136 (12.0)
[60, 70]	17 (1.5)
Marriage	
Singlehood	3 (0.2)
Married	1,113 (98.5)
Divorce	14 (1.3)
Education	
Illiterate	67 (5.9)
Literate and up to lower secondary level	158 (14.0)
Low up to middle secondary level	676 (59.8)
Higher secondary level and above	229 (20.3)
Smoking status	
No	829 (73.4)
Yes	301 (26.6)
Drinking status	
No	817 (72.3)
Yes	313 (27.7)
BLL (μg/L)	
Mean ± *SD*	386.73 ± 177.93 (17–1,060)

Note

BLL, blood lead level.

### 3.2 Association of lead exposure susceptibility with individual SNPs

The details for associations between BLL and *TRPV5* polymorphisms were summarized in Table 2. All polymorphism analyses had been adjusted for age, gender, marriage, education, smoking and drinking. In rs4252424, the distribution of CC, CT and TT genotype was 1068, 121, and 0, which was in concordance to the description of SNP database in NCBI (https://www.ncbi.nlm.nih.gov/snp/). And the rs4252424 genotypes had displayed a significant correlation with the BLL in all participants (*β* = −0.069, *p*_linear_ = 0.029). However, there was no significant association between any of the other seven SNPs and BLL. These results indicated the importance and potential use of SNP rs4252424 for lead‐susceptible population.

**Table 2:** Genotype of MALAT1 polymorphism and BLLs in participants

SNPs	Gene name	Genotypes	Numbers	MAF	*β* a	*p*_linear_ a
rs4252381	TRPV5	CC/CA/AA	1144/44/1	0.01	0.027	0.411
rs4252416	TRPV5	TT/GT/GG	1049/133/7	0.10	−0.663	0.507
rs4252499	TRPV5	GG/AG/AA	1145/44/0	0.03	0.028	0.392
rs4252402	TRPV5	CC/CT/TT	1026/159/4	0.09	−0.057	0.081
rs1568760	TRPV5	AA/AG/GG	1105/84/0	0.03	0.007	0.843
rs4236480	TRPV5	GG/AG/AA	992/197/0	0.10	−0.043	0.203
rs4252511	TRPV5	AA/AT/TT	1127/62/0	0.04	0.048	0.155
rs4252424	TRPV5	CC/CT/TT	1068/121/0	0.05	−0.069	**0.039**

0.039 < 0.05, The differences were statistically significant (in bold).

a Data were analyzed under a dominant genetic model and adjusted for age, gender, marriage, education status, smoking status, and drinking status.

**Figure 2:**
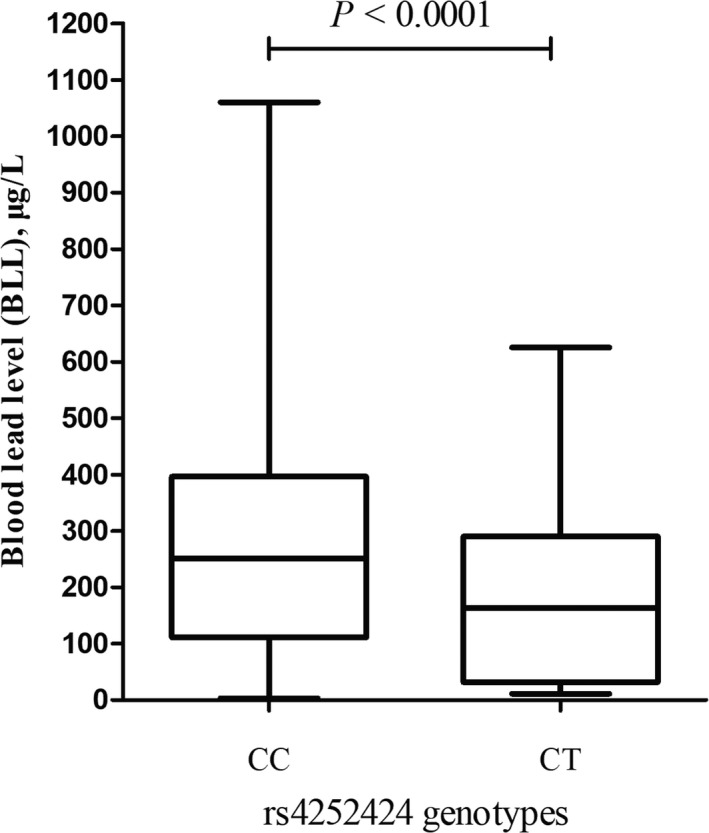
eQTL analysis of rs4252424

Based on the above results, we performed eQTL analysis of rs4252424 by contrasting the BLLs of individuals carrying CC genotype with CT. As shown in Figure 2, workers carrying the CT genotype had 30% decrease in BLL compared with those with CC genotype (*p* < 0.0001). These results also suggested the CT allele was associated with lower susceptibility to lead exposure and resulted in a lower internal lead level, when individuals underwent the exposure similar lead external dose.

## 4 DISCUSSION

In this study, we have performed genotyping of SNPs in *TRPV5* gene among the lead‐exposed workers. The notable and novel finding is that one of the eight potential SNPs, rs4252424 is significantly associated with the risk of lead exposure (measured in BLL). Workers carrying CT genotype of this SNP have been more resistant to lead exposure.

It is proposed that lead could trigger the dysregulation of calcium, which could finally threaten the population health via different calcium‐related disease. As previous reported, lead influenced calcium balance and serum calcium decreased with high BLL in children (Flora et al., 2012). It was also reported that adolescents who had been exposed to lead in their work excreted higher urinary calcium than the control group (Oktem et al., 2004). Urinary calcium is an important clinical index that measures the performance of calcium metabolism and increases when nephrolithiasis and osteoporosis occur (Kasperk & Bartl, 2014; Liebman, Taylor, & Bushinsky, 2006; Saidenberg‐Kermanac'h et al., 2014). As lead tends to increase urinary calcium, it is highly likely that the Ca^2+^ balance and skeleton system are fragile due to the wasting in the urine. Previous researches have demonstrated that TRPV5 is highly involved in the process of Ca^2+^ transport (Mensenkamp et al., 2007). Hypercalciuria, polyuria, and urinary acidification occurred in *TRPV5*‐null (*TRPV5^−^*/*^−^*) mice’ renal. Moreover, 2.9‐fold of serum1, 25(OH)_2_D_3 _was detected in *TRPV5*KO mice (Hoenderop, Leeuwen, Eerden, Kersten, Kemp et al., 2003). Likewise, increased serum PTH was found in older mice lacking *TRPV5 *(van de Graaf, Chang, Mensenkamp, Hoenderop, & Bindels, 2006). It was demonstrated that calbindin‐D28k and NCX1 were down‐regulated by TRPV5, despite increased 1,25‐(OH)_2_D_3_ levels (Hoenderop, Leeuwen, Eerden, Kersten, Kemp et al., 2003). All these studies agree that TRPV5 affects calcium wasting and is essential for Ca^2+^ re‐absorption in renal. TRPV5 may play a significant role in calcium imbalance under lead exposure.

*TRPV5* polymorphisms have been mainly documented in hypercalciuric disorders. For example, one study detected *TRPV5*‐A8V, R154H, and A561T mutants in 20 patients with renal hypercalciuria, despite no effects on channel currents (Renkema et al., 2009). S682P mutation of TRPV5 caused autosomal dominant hypercalciuria in mouse model and a lower baseline Ca^2+^ influx than wild‐type *TRPV5* in HEK293 cells (Loh et al., 2013). Another research found a *TRPV5 *polymorphism (rs4236480) was associated with stone multiplicity of calcium nephrolithiasis, as the risk of stone multiplicity increased in patients carrying TT+CT genotype than those with CC genotype (Khaleel et al., 2015). It was also reported that there was no significant difference between renal cancer and *TRPV5 *rs4252499 (Southard et al., 2012). However, A563T (rs4252499) variation in *TRPV5 *existed specifically in African Americans and increased Ca^2+^ Influx (Na et al., 2009). The potential mechanism was that amino acid residue 563 interacted with V540 and D542, which were involved in Ca^2+^ selectivity and Mg^2+^ blockade, respectively (Wang, Holmes, & Peng, 2016). In addition, A563T variation was able to alter electrostatic potential of the outer surface of the pore. In a preventive study, SNP rs4252424 distributed differently among all participants, indicating that rs4252424 was potentially associated with lead exposure. Since TRPV5 was able to influence calcium wasting (Hoenderop, Leeuwen, Eerden, Kersten, derKemp et al., 2003), it is rationally believed that SNP rs4252424 might increase urinary calcium excretion and aggravate calcium deficiency.

Limitations of this study still exist. Since the potential SNPs were selected based on published articles and public databases, we might miss some other potential functional genetic variability, which were not necessarily SNPs. Second, bias could be induced by the relatively small sample size of this study, and further studies with larger sample size are needed to validate our finding. Additionally, participants in this study were all Chinese. It is desirable to extend this study to different ethnic groups and test the validity of our finding. Moreover, although we have revealed the association between SNP rs4252424 and susceptibility to lead exposure, actual molecular and cellular mechanisms still need to be explored.

## 5 CONCLUSION

In summary, we explored the association between *TRPV5* polymorphism and lead susceptibility, and identified that SNP rs4252424 was significantly associated with lead exposure susceptibility; CT genotype of rs4252424 was more prevalent in individuals with low BLLs.

## DISCLOSURE

The authors declare that they have no conflicts of interest concerning this article.
